# Supplementary material for: Effects of Daily Consumption of an Aqueous Dispersion of Free-Phytosterols Nanoparticles on Individuals with Metabolic Syndrome: A Randomised, Double-Blind, Placebo-Controlled Clinical Trial
Source: Nutrients. 2020 Aug 10;12(8):2392. doi: 10.3390/nu12082392 (PMC7468816; doi:10.3390/nu12082392)
Supplement: Supplementary file 1 [file nutrients-12-02392-s001.pdf]

Supplementary material

Effects of daily consumption of an aqueous dispersion of free-phytosterols nanoparticles on patients with metabolic syndrome: A randomised, double-blind, placebo-controlled clinical trial

Table S1. Schedule of procedures and assessments at each visit.

Procedure/ assessment	Screening	Visit 1	Visit 2	Visit 3	Visit 4
Written informed consent		X			
Demographic information	X	X			
Health history	X	X			
Randomisation		X			
Concomitant medication	X	X	X	X	X
Physical examination		X	X	X	X
Vital signs:<br>Blood pressure, heart rate		X	X	X	X
Anthropometric measurements:<br>Height, weight, waist circumference	X	X	X	X	X
Lipid profile:<br>Total cholesterol, LDL-c, VLDL-c, HDL-c, triglycerides, lipid particles	X	X	X	X	X
Biochemical profile:<br>Insulin, fasting glycaemia	X	X	X	X	X
Glycated haemoglobin		X		X	X
Vitamin D		X	X	X	X
Pregnancy test (urine)		X			
Safety outcome evaluation			X	X	X
Bowel habit evaluation		X	X	X	X
Diet and physical activity		X	X	X	X
Participant's notebook check			X	X	X
Product delivery		X	X	X	
Phone contact	X---X				

Table S2. Distribution of participants (%) with 'abnormal' levels for each MS criterion at baseline and visit 4 by group.

	**Phytosterols (n=102)**		**Placebo (n=100)**		**Difference of proportion (in %) between groups* at V4 (95% CI)**	**p-value***
	**Baseline n (%)**	**V4 n (%)**	**Baseline n (%)**	**V4 n (%)**		
**Waist circumference**						
All 'abnormal'	102 (100)	96 (94.12)	100 (100)	97 (97)	2.88 (-2.78 - 8.54)	0.321
- Men: ≥90 cm	28 (100)	27 (96.43)	26 (100)	26 (100)	3.57 (-3.3 - 10.44)	0.331
- Women: ≥80 cm	74 (100)	69 (93.24)	74 (100)	71 (95.95)	2.7 (-4.57 - 9.98)	0.467
**Triglycerides**						
All: ≥150 mg/dL	48 (47.06)	33 (32.35)	45 (45)	48 (48)	15.65 (2.29 - 29)	0.023
- Men	19 (67.86)	14 (50)	19 (73.08)	17 (65.38)	15.38 (-10.64 - 41.41)	0.253
- Women	29 (39.19)	19 (25.68)	26 (35.14)	31 (41.89)	16.21 (1.2 - 31.23)	0.037
**HDL-c**						
All 'abnormal'	61 (59.8)	55 (53.92)	64 (64)	56 (56)	2.08 (-11.64 - 15.8)	0.767
- Men: <40 mg/dL	16 (57.14)	14 (50)	17 (65.38)	14 (53.85)	3.85 (-2.28 - 30.5)	0.778
- Women: <50 mg/dL	45 (60.81)	41 (55.41)	47 (63.51)	42 (56.76)	1.35 (-14.64 - 17.34)	0.868
**SBP** ≥130 mmHg OR **DBP** ≥85 mmHg						
All 'abnormal'	57 (55.9)	39 (38.24)	43 (43)	36 (36)	2.24 (-15.56 - 11.09)	0.742
- Men	18 (64.29)	16 (57.1)	18 (69.23)	14 (53.8)	3.3 (-29.8 - 23.22)	0.808
- Women	39 (52.7)	23 (31.1)	25 (33.78)	22 (29.7)	-1.4 (-1.62 - 13.5)	0.858
**Glycemia**						
All: ≥100 mg/dL	25 (24.51)	30 (29.41)	18 (18)	23 (23)	-6.41 (-18.5 - 5.68)	0.300
- Men	5 (17.86)	7 (25)	7 (26.92)	9 (34.62)	9.62 (-14.71 - 33.94)	0.439
- Women	20 (27.03)	23 (31.08)	11 (14.86)	14 (18.92)	-12.16 (-25.98 - 1.65)	0.088

*Difference between placebo – phytosterols using test of proportions.

Table S3. Median and 25th-75th percentiles for each secondary outcome at visits 2, 3, and 4 by group.

	Phytosterol (n=102)			Placebo (n=100)		
	**V2**	**V3**	**V4**	**V2**	**V3**	**V4**
**TChol** (mg/dL)<br>(p25-p75)<br>p-value*	187.5<br>(167-211)	195.5<br>(167-228)	193<br>(165-221)	198.5<br>(173.5-233.5)<br>0.0738	195.5<br>(173-223)<br>0.9405	195<br>(170-223)<br>0.5609
**LDL-c** (mg/dL)<br>(p25-p75)<br>p-value*	116.5<br>(94-139)	118<br>(97-143)	119.5<br>(96-138)	120.5<br>(102-147)<br>0.1193	117.5<br>(98-142)<br>0.8928	120.5<br>(102-140)<br>0.7189
**VLDL-c** (mg/dL)<br>(p25-p75)<br>p-value*	24<br>(18-41)	26.5<br>(20-36)	25<br>(18-35)	28<br>(19-40.5)<br>0.3576	27<br>(17-43)<br>0.5616	28.5<br>(18-37)<br>0.3026
**HDL-c** (mg/dL)<br>(p25-p75)<br>p-value*	44.5<br>(38-53)	46<br>(39-56)	46<br>(40-55)	44<br>(39-52)<br>0.7251	44<br>(38-51.5)<br>0.2262	45<br>(39-53.5)<br>0.6839
**TG** (mg/dL)<br>(p25-p75)<br>p-value*	120<br>(92-203)	126.5<br>(97-173)	123<br>(87-175)	144<br>(98.5-200.5)<br>0.2604	140.5<br>(87-214.5)<br>0.3698	145<br>(99.5-191.5)<br>0.1137
**FG** (mg/dL)<br>(p25-p75)<br>p-value*	91.5<br>(86-100)	92.5<br>(86-100)	95<br>(90-102)	92<br>(85.5-100)<br>0.8425	91<br>(87-99)<br>0.5911	92<br>(87-98)<br>0.1712
**HbA1c** (%)<br>(p25-p75)<br>p-value*	Not taken	5.42<br>(5.12-5.88)	5.42<br>(5.12-5.88)	Not taken	5.27<br>(5.08-5.77)<br>0.2128	5.28<br>(2.08-5.85)<br>0.2180
**FI** (μUl/mL)<br>(p25-p75)<br>p-value*	16.4<br>(10.48-20.88)	16.68<br>(12.32-24.74)	17.68<br>(11.68-23.86)	13.96<br>(10.33-23.48)<br>0.8060	15.79<br>(10.73-22.25)<br>0.2707	16.45<br>(10.9-23.36)<br>0.4995
**HOMA-IR index**<br>(p25-p75)<br>p-value*	3.65<br>(2.28-5.21)	3.83<br>(2.67-5.83)	4.07<br>(2.69-6.32)	3.21<br>(2.26-6.1)<br>0.7967	3.55<br>(2.48-5.43)<br>0.2396	3.63<br>(2.42-5.45)<br>0.2755
**Weight** (kg)<br>(p25-p75)<br>p-value*	78.75<br>(72-90.2)	79.05<br>(71.1-90)	78.5<br>(71.7-90)	79.1<br>(69.5-90)<br>0.8851	79.15<br>(69.25-90.05)<br>0.6823	80.05<br>(68.75-90.1)<br>0.6859
**BMI** (kg/mt^2)<br>(p25-p75)<br>p-value*	30.81<br>(27.32-33.86)	30.6<br>(27.38-34.02)	30.27<br>(27.43-34.16)	30.99<br>(28.16-34.49)<br>0.4497	30.82<br>(28.29-34.99)<br>0.3368	30.94<br>(28.11-34.5)<br>0.3497
**WC** (cm)<br>(p25-p75)<br>p-value*	97<br>(92-106)	97<br>(91-103)	94<br>(89-102)	98.5<br>(93-108)<br>0.1864	100<br>(93-108.5)<br>0.0154	99<br>(93-109.5)<br>0.0022
**SBP** (mm Hg)<br>(p25-p75)<br>p-value*	120.5<br>(112-130)	121.5<br>(111-129)	119<br>(112-129)	119.5<br>(111.5-129.5)<br>0.6095	118.5<br>(111-131)<br>0.9042	119<br>(112.4-128)<br>0.9242
**DBP** (mm Hg)<br>(p25-p75)<br>p-value*	82<br>(74-89)	79<br>(74-87)	79.5<br>(73-87)	80<br>(74-85)<br>0.1214	81<br>(75.5-87)<br>0.4408	80<br>(72.5-86)<br>0.6820

*p-value between phytosterol and placebo groups at V2, V3, and V4.

TChol: total cholesterol; LDL-c: low-density lipoprotein cholesterol; VLDL-c: very-low-density lipoprotein cholesterol; HDL-c: high-density lipoprotein cholesterol; TG: triglycerides; HbA1c: glycated haemoglobin; FI: fasting insulin; BMI: body mass index; WC: waist circumference; SBP: systolic blood pressure; DBP: diastolic blood pressure.

Table S4. Absolute difference between V2 and V1, V3 and V1, and V4 and V1 for each secondary outcome by group. All data are presented as median and 25th-75th percentiles.

	Phytosterol (n=102)			Placebo (n=100)		
	A Diff V2-V1	A Diff V3-V1	A Diff V4-V1	A Diff V2-V1	A Diff V3-V1	A Diff V4-V1
**TChol** (mg/dL)<br>(p25-p75)<br>p-value*	-6<br>(-16 - 4)	-1<br>(-13 - 14)	-3.5<br>(-20 9)	0<br>(-14.5 - 16.5)<br>0.0131	0<br>(-19.5 - 14)<br>0.9443	-1<br>(-18 - 12.5)<br>0.4206
**LDL-c** (mg/dL)<br>(p25-p75)<br>p-value*	-3.5<br>(-15 - 7)	-1<br>(-14 - 18)	-2<br>-15 11	0<br>(-14 - 10)<br>0.2027	-2.5<br>(-15 - 7.5)<br>0.5017	-1.5<br>(-15 - 8.5)<br>0.8510
**VLDL-c** (mg/dL)<br>(p25-p75)<br>p-value*	-1.5<br>(-8 - 3)	-1.5<br>(-8 - 4)	-3<br>(-11 - 4)	1<br>(-6.5 - 7.5)<br>0.0438	0<br>(-8 - 7.5)<br>0.1983	0<br>(-8 - 6)<br>0.1157
**HDL-c** (mg/dL)<br>(p25-p75)<br>p-value*	0<br>(-1 - 3)	2<br>(-1 - 7)	1<br>(-1 - 5)	1<br>(-2 - 3.5)<br>0.8943	1<br>(-2 - 4.5)<br>0.1083	1<br>(-2 - 5)<br>0.6563
**TG** (mg/dL)<br>(p25-p75)<br>p-value*	-9.5<br>(-42 - 14)	-13<br>(-46 - 17)	-16.5<br>(-57 - 15)	5<br>(-30.5 - 37)<br>0.0138	0.5<br>(-41.5 - 34.5)<br>0.0648	1.5<br>(-40 - 32)<br>0.0245
**FG** (mg/dL)<br>(p25-p75)<br>p-value*	-1<br>(-6 - 6)	0<br>(-4 - 5)	2<br>(-3 - 7)	2<br>(-4 - 6)<br>0.0467	1<br>(-4 - 6)<br>0.5404	1.5<br>(-3 - 6)<br>0.9769
**HbA1c** (%)<br>(p25-p75)<br>p-value*	Not taken	0.075<br>(-0.1 - 0.31)	0.2<br>(0.01 - 0.44)	Not taken	0.095<br>(-0.11 - 0.21)<br>0.8069	0.13<br>(0.01 - 0.43)<br>0.3653
**FI** (µUl/mL)<br>(p25-p75)<br>p-value*	0.64<br>(-2.72 - 2.87)	1.15<br>(-2.17 - 5.8)	2.46<br>(-1.33 - 5.16)	1.34<br>(-2.95 - 6.21)<br>0.0848	2.28<br>(-1.4 - 6.2)<br>0.3875	2.67<br>(-1.1 - 5.48)<br>0.8135
**HOMA-IR index**<br>(p25-p75)<br>p-value*	0.01<br>(-0.87 - 0.63)	0.31<br>(-0.48 - 1.67)	0.62<br>(-0.25 - 1.46)	0.38<br>(-0.66 - 1.63)<br>0.0233	0.45<br>(-0.22 - 1.42)<br>0.4172	0.57<br>(-0.19 - 1.32)<br>0.9578
**Weight** (kg)<br>(p25-p75)<br>p-value*	0<br>(-1.1 - 1)	-0.15<br>(-1.6 - 1.3)	-0.2<br>(-2.1 - 1.4)	-0.15<br>(-1.2 - 0.9)<br>0.6378	0<br>(-0.8 - 1.55)<br>0.2696	0.4<br>(-0.95 - 1.55)<br>0.0905
**BMI** (kg/mt^2)<br>(p25-p75)<br>p-value*	0<br>(-0.35 - 0.34)	-0.06<br>(-0.67 - 0.55)	-0.07<br>(-0.81 - 0.61)	-0.06<br>(-0.46 - 0.31)<br>0.5329	0<br>(-0.34 - 0.62)<br>0.2922	0.15<br>(-0.33 - 0.62)<br>0.1211
**WC** (cm)<br>(p25-p75)<br>p-value*	-1<br>(-4 - 1)	-2<br>(-5 - 0)	-4<br>(-7 - -1)	-0.75<br>(-3 - 1)<br>0.1666	0<br>(-2 - 2)<br>0.0011	0<br>(-3 - 2.5)<br>0.0001
**SBP** (mm Hg)<br>(p25-p75)<br>p-value*	-3.5<br>(-11 - 4)	-2.5<br>(-10 - 6)	-2<br>(-12 - 5)	-1<br>(-9 - 4)<br>0.4611	-0.5<br>(-10 - 5.5)<br>0.5062	-1<br>(-10.5 - 7.5)<br>0.2753
**DBP** (mm Hg)<br>(p25-p75)<br>p-value*	-2<br>(-8 - 3)	-2<br>(-9 - 3)	-2.5<br>(-9 - 3)	-2<br>(-9.5 - 2.5)<br>0.5382	-2<br>(-8 - 3.5)<br>0.6231	-3<br>(3.5 - -10)<br>0.9099

*p-value between phytosterol and placebo groups.

TChol: total cholesterol; LDL-c: low-density lipoprotein cholesterol; VLDL-c: very-low-density lipoprotein cholesterol; HDL-c: high-density lipoprotein cholesterol; TG: triglycerides; HbA1c: glycated haemoglobin; FI: fasting insulin; BMI: body mass index; WC: waist circumference; SBP: systolic blood pressure; DBP: diastolic blood pressure

Table S5. Relative difference (%) between V2 and V1, V3 and V1, and V4 and V1 for each secondary outcome by group. All data are presented as median and 25th-75th percentiles.

	Phytosterol (n=102)			Placebo (n=100)		
	R Diff V2-V1	R Diff V3-V1	R Diff V4-V1	R Diff V2-V1	R Diff V3-V1	R Diff V4-V1
**TChol**<br>(p25-p75)<br>p-value*	-2.99<br>(-8.33 - 2.37)	-0.5<br>(-6.74 - 7.1)	-1.78<br>(-9.19 - 4.74)	0<br>(-7.27 - 8.89)<br>0.0120	0<br>(-9.62 - 8.39)<br>0.9722	-0.68<br>(-8.72 - 7.42)<br>0.3718
**LDL-c**<br>(p25-p75)<br>p-value*	-2.74<br>(-12.5 - 6.02)	-0.98<br>(-12 - 12.1)	-1.44<br>(-10.91 - 10.34)	0<br>(-11.44 - 10.31)<br>0.1764	-1.84<br>(-12.1 - 7.94)<br>0.6183	-0.94<br>(-11.37 - 8.7)<br>0.9376
**VLDL-c**<br>(p25-p75)<br>p-value*	-5.56<br>(-26.1 - 15.4)	-7.69<br>(-28.20 - 15.79)	-10.52<br>(-35.9 - 19.05)	3.52<br>(-19.44 – 25)<br>0.0263	0<br>(-25.54 - 31.03)<br>0.1883	0<br>-21.28 - 62.15)<br>0.0381
**HDL-c**<br>(p25-p75)<br>p-value*	0<br>(-2.86 - 7.84)	5.33<br>(-2.70 - 14.47)	2.53<br>(-2.5 - 12.28)	1.82<br>(-4.77 - 7.9)<br>0.9625	2.17<br>(-4.6 - 10.87)<br>0.0770	2.56<br>(-5.20 - 10.07)<br>0.5077
**TG**<br>(p25-p75)<br>p-value*	-7.98<br>(-27.78 - 13.64)	-9.68<br>(-28.54 - 14.15)	-12.55<br>(-38.14 - 9.4)	3.9<br>(-18.9 – 24)<br>0.0151	.34<br>(-25.62 - 30.49)<br>0.0760	0.81<br>(-21.14 - 26.2)<br>0.0061
**FG**<br>(p25-p75)<br>p-value*	-1.16<br>(-6.5 - 6.52)	0<br>(-4.35 - 6.25)	1.88<br>(-3.45 – 8)	2.21<br>(-4.16 - 7.59)<br>0.0512	1.09<br>(-4.30 - 6.77)<br>0.4553	1.7<br>(-3.25 - 6.89)<br>0.9290
**HbA1c**<br>(p25-p75)<br>p-value*	Not taken	1.42<br>(-1.73 - 5.81)	3.95<br>(0.19 - 8.83)	Not taken	1.75<br>(-2.05 - 4.34)<br>0.8833	2.6<br>(0.16 - 8.4)<br>0.3763
**FI**<br>(p25-p75)<br>p-value*	3.04<br>(-13.71 - 20.79)	10.26<br>(-11.92 - 46.67)	17.6<br>(-6.71 - 45.63)	17.63<br>(-18.7 - 45.81)<br>0.0648	21.04<br>(-6.66 - 50.59)<br>0.2950	22.5<br>(-7.85 - 52.91)<br>0.5881
**HOMA-IR index**<br>(p25-p75)<br>p-value*	0.15<br>(-22 - 28.29)	9.81<br>(-16.7 - 55.8)	19.45<br>(-7.51 - 53.73)	14.7<br>(-20.03 - 53.26)<br>0.0211	22.65<br>(-7.32 - 52.04)<br>0.3062	25.12<br>(-6.48 - 57.52)<br>0.6353
**Weight**<br>(p25-p75)<br>p-value*	0<br>(-1.11 - 1.07)	-0.20<br>(-2.09 - 1.69)	-0.19<br>(-2.05 – 2)	-0.19<br>(-1.59 - 1.01)<br>0.5401	0<br>(-1.06 - 2.06)<br>0.3465	0.44<br>(-1.04 - 1.99)<br>0.1453
**BMI**<br>(p25-p75)<br>p-value*	0<br>(-1.11 - 1.07)	-0.20<br>(-2.09 - 1.69)	-0.19<br>(-2.05 – 2)	-0.19<br>(-1.59 - 1.01)<br>0.5401	0<br>(-1.06 - 2.06)<br>0.3465	0.44<br>(-1.04 - 1.99)<br>0.1453
**WC**<br>(p25-p75)<br>p-value*	-1.03<br>(-3.57- 0.91)	-2.09<br>(-5.21 - 3.39)	-3.74<br>(-7.5 - 1.01)	-0.72<br>(-2.7 - 1.12)<br>0.1496	0<br>(-2.2 - 2.21)<br>0.0007	0<br>(-2.95 - 2.34)<br>0.0000
**SBP**<br>(p25-p75)<br>p-value*	-2.56<br>(-8 - 3.38)	-2.24<br>(-8 - 4.42)	-1.65<br>(-8.96 - 3.65)	-0.86<br>(-7.5 - 3.61)<br>0.5087	-0.38<br>(-7.98 - 4.62)<br>0.4972	-0.92<br>(-8.13 - 6.27)<br>0.2792
**DBP**<br>(p25-p75)<br>p-value*	-2.33<br>(-8.89 - 3.75)	-2.47<br>(-12.12 - 3.61)	-3.19<br>(-10.98 - 3.45)	-3.06<br>(-10.16 - 3.54)<br>0.5465	-2.59<br>(-9.81 - 4.17)<br>0.6064	-3.57<br>(-12.9 - 3.83)<br>0.9319

*p-value between phytosterol and placebo groups.

TChol: total cholesterol; LDL-c: low-density lipoprotein cholesterol; VLDL-c: very-low-density lipoprotein cholesterol; HDL-c: high-density lipoprotein cholesterol; TG: triglycerides; HbA1c: glycated haemoglobin; FI: fasting insulin; BMI: body mass index; WC: waist circumference; SBP: systolic blood pressure; DBP: diastolic blood pressure

Table S6. Baseline characteristics of participants in the sub-sample to evaluate the particle number for lipoproteins by group. All data are presented as median and 25th-75th percentiles.

	**Phytosterol (n=59)**	**Placebo (n=57)**
**Sociodemographic variables**		
Age in months, median (p25-p75)	509 (416-630)	542 (418-631)
Sex		
Men, n (%)	19 (32.2)	19 (33.33)
Women, n (%)	40 (67.80)	38 (66.67)
Socioeconomic status (self-reported)		
High, n (%)	3 (5.08)	2 (3.51)
Middle, n (%)	56 (94.92)	55 (96.49)
Low, n (%)	0	0
**General health-related information**		
Underlying health condition		
Yes, n (%)	68(13.56)	11 (19.30)
No, n (%)	51 (86.44)	46 (80.70)
Concomitant medication		
Yes, n (%)	25 (42.37)	26 (45.61)
No, n (%)	34 (57.63)	31 (54.39)
Smoking		
Yes, n (%)	29 (49.15)	35 (61.40)
No, n (%)	30 (50.85)	22 (38.60)
Alcohol consumption		
Yes, n (%)	18 (30.51)	21 (36.84)
No, n (%)	41 (69.49)	36 (63.16)
Physical activity		
Yes, n (%)	31 (52.54)	31 (54.39)
No, n (%)	28 (47.46)	26 (45.61)
**Blood test parameters**, median (p25-p75)		
Total cholesterol (mg/dL)	196 (170-220)	191 (163-214)
LDL-c (mg/dL)	116 (99-141)	114 (98-133)
VLDL-c (mg/dL)	28 (21-42)	28 (18-42)
Insulin (µUl/mL)	16.32 (10.44-24.11)	12.63 (8.86-19.39)
Glycated haemoglobin - HbA1c (%)	5.42 (5.07-6.07)	5.35 (5.01-5.76)
Vitamin D (ng/mL)	24.5 (20.2-28.8)	23.3 (18.6-29.2)
**Anthropometric measurements and vital signs**, median (p25-p75)		
Weight (kg)	82.9 (77.3-93.7)	83.8 (71-95)
Height (mt)	1.6 (1.55-1.68)	1.61 (1.55-1.71)
Body mass index (kg/mt^2)	32.18 (28.68-35.21)	30.46 (28.59-34.31)
Heart rate (beats per minute)	72 (67-78)	71 (65-75)
**Metabolic syndrome variables**, median (p25-p75)		
Waist circumference (cm)	101 (96-110)	103 (94-111)
Triglycerides (mg/dL)	140 (108-221)	141 (92-208)
HDL-c (mg/dL)	42 (37-52)	43 (37-50)
Systolic blood pressure (mm Hg)	127 (118-137)	123 (113-138)
Diastolic blood pressure (mm Hg)	86 (80-94)	84 (76-90)
Fasting glycaemia (mg/dL)	95 (89-106)	92 (86-99)
**Lipoproteins at baseline**, median (p25-p75)		
LDL-p (nmol/L)	806.3 (695.1-917.3)	819.2 (719.6-933.73)
Small LDL-p (nmol/L)	444.4 (408.9-508.21)	472.17 (417.5-550)
Medium LDL-p (nmol/L)	225.8 (174.8-283.3)	224.2 (177.01-272.5)

Large LDL-p (nmol/L)	114.5 (94.11-124.6)	115.8 (99.79-130.7)
VLDL-p (nmol/L)	52.07 (35.6-85.8)	53.58 (34.41-78.71)
Small VLDL-p (nmol/L)	45.62 (29.06-73.29)	46.38 (29.65-68.86)
Medium VLDL-p (nmol/L)	5.59 (4.09-8.17)	5.68 (3.81-7.68)
Large VLDL-p (nmol/L)	1.29 (0.89-1.94)	1.27 (0.95-1.9)
HDL-p (μmol/L)	25.3 (22.03-28.3)	24.64 (20.6-28.3)
Small HDL-p (μmol/L)	16.52 (13.29-18.37)	16.51 (13.46-19.33)
Medium HDL-p (μmol/L)	8.45 (7.71-9.35)	8.19 (6.96-8.65)
Large HDL-p (μmol/L)	0.27 (0.24-0.3)	0.25 (0.23-0.3)

(p25-p75): 25th percentile and 75th percentile; LDL-c: low-density lipoprotein cholesterol; VLDL-c: very-low-density lipoprotein cholesterol; HDL-c: high-density lipoprotein cholesterol; VLDL-p: very-low-density lipoprotein particles; LDL-p: low-density lipoprotein particles; HDL-p: high-density lipoprotein particles.

Table S7. Median and 25th-75th percentiles for each lipoprotein at visits 2, 3, and 4 by group.

	Phytosterol (n=59)			Placebo (n=57)		
	V2	V3	V4	V2	V3	V4
**LDL-p** (nmol/L) (p25-p75)	803.5 (704.9-881.5)	831.6 (732.4-974.3)	880.8 (775.7-1024)	859.7 (718.5-1010)	908.3 (792-1007)	916.8 (791.5-1016)
**s-LDL-p** (nmol/L) (p25-p75)	459.8 (394.2-504)	475.1 (419.5-536.7)	530.2 (442.4-619.9)	492.6 (420-585.2)	515.8 (433.6-577.1)	542.3 (451.8-611.9)
**m-LDL-p** (nmol/L) (p25-p75)	227.5 (167-291.1)	235.5 (203.6-305.2)	240.4 (185.7-295.2)	227.8 (184.2-276)	251.5 (205.5-294.2)	256.15 (201.7-288.6)
**l-LDL-p** (nmol/L) (p25-p75)	114.7 (98.5-128.4)	116.1 (96.9-130.8)	115.2 (95.8-130.8)	109.2 (99.2-131.6)	118.5 (102-132.6)	114.9 (100.6-133.3)
**VLDL-p** (nmol/L) (p25-p75)	44.54 (31.6-71.2)	48.3 (38-64.7)	46.45 (33.6-75.6)	59.1 (37.3-82.8)	47.71 (29.5-74.2)	55.6 (32.2-73.3)
**s-VLDL-p** (nmol/L) (p25-p75)	38.36 (26.5-62)	40.63 (31.8-55.4)	40.59 (28.4-66.6)	53.55 (32-73.5)	42.11 (24.8-65)	49.66 (27.2-65.6)
**m-VLDL-p** (nmol/L) (p25-p75)	5.05 (4-7.7)	5.53 (4.5-7.6)	4.97 (4.1-7.5)	5.86 (4.3-7.7)	4.91 (3.8-7.55)	5.11 (4.1-6.5)
**l-VLDL-p** (nmol/L) (p25-p75)	1.15 (0.9-1.7)	1.15 (0.9-1.5)	1.12 (0.1-1.7)	1.36 (1.1-2)	1.16 (0.8-1.8)	1.37 (0.9-1.6)
**HDL-p** (µmol/L) (p25-p75)	23.75 (21.6-26.9)	24.57 (21.8-27.1)	23.91 (21.2-26.1)	23.84 (21.3-27)	22.7 (20.9-26.8)	23.45 (21.5-25.5)
**s-HDL-p** (µmol/L) (p25-p75)	15.93 (13.5-18)	16.27 (14.4-18.5)	15.51 (13.1-17.6)	16 (13.4-17.9)	15.5 (13.3-18.4)	15.55 (13.2-17.1)
**m-HDL-p** (µmol/L) (p25-p75)	7.93 (7.19-8.91)	8.06 (6.99-9.03)	7.96 (7.1-8.58)	7.68 (7.16-8.52)	7.78 (7.03-8.51)	7.92 (7.12-8.41)
**l-HDL-p** (µmol/L) (p25-p75)	0.25 (0.23-0.29)	0.26 (0.23-0.29)	0.25 (0.23-0.28)	0.25 (0.23-0.29)	0.25 (0.23-0.28)	0.25 (0.22-0.28)
